# Onosmodium Virginianum*Prepared for the Kansas State Homœopathic Society.

**Published:** 1893-07

**Authors:** W. A. Yingling

**Affiliations:** Nonchalanta, Kan.


### ONOSMODIUM VIRGINIANUM.*

W. A. Yingling, M. D., Nonchalanta, Kan.

To the homœopathic physician a new remedy, well-proven, is an acquisition of greater importance than honor or wealth, for his sole duty being to relieve the sufferings of humanity, he acquires a new tool with which to accomplish his work. To the degree that the new remedy has peculiar characteristics its

---

* Prepared for the Kansas State Homœopathic Society.

value is enhanced, to the extent that the pathogenetic effects are different from every other drug its usefulness becomes the more apparent. Generalities constitute a poor basis upon which to prescribe. Peculiarities, the unusual symptoms, give certainty and assurance in every prescription.

We have in *Onosmodium* a remedy with some peculiarities, and occupying a sphere unique, a curative range differing from that of every other drug. The remedy holds within its grasp the power to restore peace to the disrupted family, and to prevent the truant husband seeking the sweets of "stolen waters" by restoring the wife to the enjoyable performance of her wifely functions, and thus gratifying the dissatisfied husband. This generation of one-child families, Malthusian, with the long train of misery entailed upon the licensed family, adultery consequent upon preventive measures, *malum in se*, has its remedy in *Onosmodium* to a very large extent.

We pass to consider the more important pathogenesis of the remedy in regular course. A great part of this paper is necessarily based upon the notes of the original author, Dr. W. E. Green, with some isolated symptoms from the journals and my own experience.

We find marked in the mental sphere a DROWSINESS OF MIND and CONFUSION OF THOUGHT, DULLNESS OF INTELLIGENCE, a DAZED feeling of the mind. The party wants to think and not move, so absorbed in thought as to forget all else and where she is. There is a *complete listlessness and apathy* of the mind; she cannot *concentrate* her thoughts. From this want of concentration there follows an impairment of the memory, *she cannot remember what is said.* In conversation she will forget the subject, will begin a new one, and then suddenly change to another. There is great *confusion of ideas.* This listlessness is so great as to cause forgetfulness of what one is reading, or that one is reading at all; the book drops in vague and listless thought. The time passes too slowly, and minutes seem like hours. There is great irritability of temper.

There is a continuous and ever-present feeling of heaviness of the head. PAINS IN THE LEFT SIDE OF THE HEAD and

*over the left eye,* extending round the left side to the back of the head and neck, greatly aggravated by moving or jarring. Intense pain driving her to bed; relieved by sleep, but soon returning after waking. There is a constant dull headache, chiefly centered over the left eye and in the left temple; always worse in the dark and when lying down. Here we have a contradictory symptom—always worse lying down. The general symptoms are ameliorated by lying down. This peculiar feature is also seen in some of the polycrests. *Bryonia-alb.* has a "pain and pressure in the shoulder when at rest." *Rhus-tox.* has a "stiff neck, with painful tension when moving;" *Arsenicum-alb.* has a headache relieved by cold water.

*Onosmodium* has a DULL, HEAVY PAIN IN THE occiput pressing upward WITH A DIZZY SENSATION. Pain changing from the right frontal eminence to the left and remaining there. Darting and throbbing in the left temple. A dull pain in the mastoid process. She cannot bear to move. A sense of fullness in the head. Relieved by eating and sleep.

The eyes are HEAVY AND DULL; the eyes feel as though one had lost a great deal of sleep. The lids are heavy. The eyeballs have a *dull, heavy pain with soreness.* A sensation of the eyes being very wide open, with a desire to look at distinct objects, it being disagreeable to look at near objects. Distant objects look very large. *Picric-acid* patients can only see clearly at very close range, often at only five inches from the eye; Natrum-sulph. has impairment of vision for distant objects. With *Onosmodium* the ocular muscles feel tense, tired, and drawn. Pains in and over left eye. Pain in upper portion of left orbit, with a feeling of expansion. The vision is impaired and blurred.

The hearing is impaired. There is a stuffed-full feeling in the ears as after catching cold. Singing in the ears as from Quinine, but very slight.

The NOSE FEELS DRY. There is a stuffed feeling in the posterior nares. The discharge from the posterior nose is whitish and sticky, producing a constant hawking. Constant sneezing in the morning; sneezing when first getting up. The bones of the nose pain.

Flushed face, with relief from the headache. That dry feeling of the nose is also present in the mouth and lips. Bitter, clammy taste in the mouth. Saliva is very scant, with the dry feeling in the mouth; cold water relieves. Sore throat. It hurts to swallow or speak. That dryness follows down the *throat* and *pharynx*, and is accompanied with *severe soreness*. Raw, scraping feeling in the throat. When swallowing the pharynx feels constricted. All the throat symptoms are relieved by cold drinks and by eating. The voice is husky. The chest feels sore.

Morning sickness like that of pregnancy. Distaste for water, yet there is a *craving for ice water and cold drinks; wants to drink often*. The abdomen *feels bloated* and distended, which is relieved by undressing. The pains in the lower part of the abdomen are also relieved by undressing or by lying on the back. This amelioration from undressing is observed to run through all the symptoms of the drug. A constant feeling as though diarrhœa would come on.

The stools are yellow, mushy, or greenish-yellow, stringy, mushy, with tenesmus. Also slimy, bloody, stringy stool with tenesmus. The provers were hurried out of bed in the morning to stool.

The urine is scanty, highly colored, dark straw and brown, very acid, and of high specific gravity. The desire is seldom, or else frequent, with scanty flow.

In regard to the sexual organs we quote from that racy writer, Dr. S. A. Jones, who says: "*Onosmodium virginianum* in its primary action seems directly opposite to *Picric-acid*. Perhaps provings of it with smaller doses will oblige me to change this *dictum*. If they do not, then *Onosmodium* will occupy the singular position of a remedy that *primarily depresses the sexual appetite*. If this should ultimately prove to be the case, it will invest this remedy with an unmistakable significance to physicians who are practicing at the *tail end* of the nineteenth century, for, from our habits of life, it is the *end* that is showing signs of distress. In estimating the validity of this suggestion, the reader will bear in mind Hahnemann's

*dictum* that *only the primary symptoms of a drug afford the indications for its therapeutical application.* This is a canon of Hahnemannian Homœopathy, and it *is true as regards the infinitesimal dose.* Then, this being true (for I will not stop to discuss it), *Picric-acid* will be indicated for the *initial stage* of sexual debility, and *Onosmodium* for the *fully developed consequences* of sexual abuse; and this, because the said 'initial stage' is characterized by erethism while the ulterior consequences are denoted by atony and asthenia. The erethism of sexual debility is plainly evinced in *Picric-acid,* and the ultimate asthenia is as really discovered in *Onosmodium virginianum.*"

In the male we find diminished sexual desire. Cold feeling in the glans penis. Nocturnal emissions. Too speedy emissions. Deficient erections with diminished pleasure.

In the female we find SEVERE UTERINE PAINS. BEARING-DOWN PAINS IN THE UTERINE REGION. Uterine cramps. *Soreness in region of uterus,* increased by *pressure* of the hand or of the clothing; had to remove the corset. Return of old uterine pains. Dull, heavy aching, and slowly pulsating pains in the ovaries. Pains pass from one ovary to the other and leave a soreness which remains till the pain returns. Ovarian pains increased by pressure. SEXUAL DESIRE COMPLETELY DESTROYED. This sympton I have verified a number of times, and in every case the parties prevented conception. The uterine pains are all better when undressed or lying on the back. Constant feeling as though the menses would appear. Menses early and profuse, but otherwise normal so far as known. Leucorrhœa light yellowish, slightly offensive and excoriating; profuse, running down the legs. Itching of the vulva aggravated by scratching and from the leucorrhœal discharge. Aching in both breasts, but worse in the left. Breasts feel swollen and engorged. Left breast feels bruised and painful on pressure. Nipples itch. In one case where this remedy was given for dryness of the nose and throat, the diminutive, almost absent, breasts were restored to their pristine glory, and resulted in the displacement of the cotton batting pads to the exceeding joy and delight of the proud woman.

*Pains in the neck,* running back from the forehead. *Dull aching in the neck.* Bearing-down pain in the lumbar region. Dull, aching pain in the lumbar region. In the female provers there was produced a pain over the crest of the left ilium. TIRED, WEARY, AND NUMB FEELING IN THE LEGS AND POPLITEAL SPACES. FEELING OF NUMBNESS, MOSTLY BELOW THE KNEES. The legs feel as if they were partially anæsthetized. The tendons and joints of the knees have a dull, aching pain. Tremulousness of the legs. DISTURBANCE OF THE GAIT IN WALKING, WITH A SENSE OF INSECURITY IN STEP. STAGGERING GAIT, *he cannot keep in the walk.* The sidewalks seem too high; he must step high which jars him and greatly aggravates the headache. Dull, heavy pain in the instep of the left foot. Numb, tingling pain in the outer side of both little toes. THE LEGS FEEL TIRED, *as though they would not sustain the weight of the body.* Sensation of formication in the calves of the legs. Ankles swollen.

*Pain in the left scapular region,* confined to a small spot. *Fluoric-acid* and *Lilium-tig.* have pain confined to a small spot in any location, while *Oxalic-acid* has a pain confined to small longitudinal spots. *Magnesia-phos.* has a sharp, burning pain, about an inch in diameter, under the border of the left scapula, as from a hot iron (see also *Phos.*); with *Onosmodium* there is a dull, aching pain in the biceps muscle, also a pain of like nature in the elbow joint and wrists. *The arms and hands feel tired and weak;* they tremble. Inability to co-ordinate the muscular movements of the arms. Pain in the phalangeal articulation.

The aggravations are generally from motion or jarring; from pressure or tightness of clothing.

The ameliorations are peculiar and marked. Better when quiet, *when lying down on the back, when undressed,* when in the open air, from sleep, *from cold drinks, from eating.*

In the generalities we find great MUSCULAR WEAKNESS OR PROSTRATION AND TIRED FEELING OVER THE ENTIRE BODY. A feeling as though one had just gotten up from a severe spell of sickness. Nervous trembling as if from hunger. The least exertion produces a tremulousness. *The muscles feel treacherous and unsteady as though one did not dare to trust them.* A desire to change position without any definite cause or reason, and without any change for the better or worse. Later in the proving there was a desire to lie down and be quiet, with a drowsy, sleepy feeling. *A sensation as if a chill would come on ;* a tired, aching, stretching, gaping, disagreeable feeling. All sensations are worse in the left side.

In my own experience I have used the remedy from the mother tincture up. I got no results from the tincture. Hardly any from the 30th, but a marked, decided, and very rapid action from the CM. I use nothing lower than the CM, and prefer the higher.

